# Antihypertensive Property of Celery: A Narrative Review on Current Knowledge

**DOI:** 10.1155/2024/9792556

**Published:** 2024-03-12

**Authors:** Sondos Alobaidi, Eman Saleh

**Affiliations:** ^1^Nutrition and Food Technology Department, Agriculture School, The University of Jordan, Amman, Jordan; ^2^Clinical Nutrition and Dietetics Department, Allied Medical Sciences College, Applied Science Private University, Amman, Jordan

## Abstract

**Background:**

The incidence of hypertension is increasing significantly on a global scale, and it is considered the leading cause of heart disease and death. Despite the availability of hypotensive drugs, they have many side effects that decrease adherence to treatments and lead to uncontrolled blood pressure. Studies have revealed that celery contains bioactive compounds that oppose hypotensive effect.

**Methods:**

A thorough literature review was conducted using Scopus, PubMed, and Google Scholar databases. To identify relevant studies on the topic, our search strategy employed keywords such as “celery,” “*Apium gravenols L*,” “hypertension,” “high blood pressure,” “apigenin,” “antihypertensive,” and “hypotensive.” The search was limited to articles published between January 2013 and December 2023. The inclusion criteria were original research articles that involved both animal and human subjects, published in English, and reported results applicable to the subject of this review. Review articles or articles in the form of theses or books were excluded.

**Results:**

The available evidence revealed that celery enhances blood pressure parameters. Clinical trials clarified that celery possesses its effect through many bioactive compounds, specifically 3-n-butylphthalide and apigenin. Based on animal and human studies, celery seems to elicit blood pressure regulation mainly by the vasodilatory, diuretic, and calcium channel-blocking properties. Furthermore, celery seed extract seems to exert a bradycardia effect.

**Conclusion:**

The current literature review showed a considerable number of studies on the hypertensive models, which confirmed that celery and its extracts are effective hypotensive agents. Some limitations in comparing published data should be considered, including differences in doses, extracts, species of celery, and administration form.

## 1. Introduction

The incidence of hypertension is increasing significantly on a global scale, and it is considered the main cause of cardiovascular disease (CVD) [1]. While CVD is the primary cause of death globally, it is expected to account for 29% of deaths by 2025. The danger of hypertension lies in its silent symptoms, which cause a variety of complications and morbidities [2]. The World Health Organization (WHO) specifies high blood pressure in measurements equal to and above 140/90 mmHg [3]. Many hypotensive medications control and treat high blood pressure, including diuretics, vasodilators, calcium channel blockers, renin inhibitors, and angiotensin-converting enzyme inhibitors [4]. Despite the availability of these drugs, they have many side effects, such as edema, muscle cramps, skin rashes, vomiting, and kidney failure [3]. Thus, side effects decrease the adherence of hypertensive patients to their treatment, which leads to uncontrolled blood pressure [4]. Populations' growing acceptance of alternative medicine and herbal plants has increased, especially among developing countries [1]. Approximately 80% of the population accepted this approach to treatment because it is safer and less expensive, has fewer side effects, and is more compatible with human bodies [5].

Celery (Apium graveolens), a biennial herb native to the Mediterranean region, has been cultivated for centuries. For over 200 years, it has been grown for medicinal purposes before being consumed as a food ingredient [6]. Celery is characterized by its exceptional yield and high resistance to disease. Studies have revealed that celery is abundant in vitamins, minerals, phthalides, and flavonoids like apigenin and luteolin, which have strong anti-inflammatory and antitumor properties. Additionally, celery contains silica, chlorophyll, and high fiber content and comprises approximately 95% water [6]. Because of its antioxidant and potential anti-inflammatory properties, different parts (seeds, leaves, stems, and roots) have been used in traditional remedies to treat arthritis, urinary tract diseases, and high blood pressure. Furthermore, it has a protective effect against CVD, high lipid profile, elevated blood sugar readings in type 2 diabetic patients, and obesity [6–8].

The extraction of bioactive compounds from celery can be influenced by the plant part and method used. Unfortunately, the use of phenolic compounds is restricted due to issues with low bioavailability, solubility, stability, and imprecise release [6, 9]. However, recent advancements in nanoencapsulation lipid-based methods are aimed at enhancing the targeted delivery of these functional compounds. Cutting-edge technologies, such as lipid-based nanoparticles, nanocrystals, and solubility-enhancing agents, such as cyclodextrin, have shown promise in improving the effectiveness of celery compounds [1, 10].

Active components of celery, including 3-n-butylphthalide (NBP) and apigenin, can help lower blood pressure by acting as diuretics and vasodilators. Additionally, their effectiveness is similar to that of calcium channel blocker drugs [11, 12]. They can also decrease cholesterol levels and arterial plaque formation, contributing to lower blood pressure. Unlike typical diuretic drugs, NBP and apigenin do not disrupt the balance between sodium and potassium levels in the blood [13]. In traditional Chinese and Indonesian societies, celery has been used to treat and control high blood pressure for a long time with effective results because of its safety and affordability, without side effects [10, 11]. Therefore, the management of hypertension can be achieved pharmaceutically and nonpharmaceutically using herbal plants such as celery [14]. Thus, the purpose of this review is to assess the impact of celery on lowering blood pressure in individuals with hypertension. The review summarized the research, providing information on the study design, sample size, intervention protocol, and efficiency of the celery intervention in reducing blood.

## 2. Methods and Search Strategy

A thorough literature review was conducted using Scopus, PubMed, and Google Scholar databases. To identify relevant studies on the topic, our search strategy employed keywords such as “celery,” “*Apium gravenols L*,” “hypertension,” “high blood pressure,” “apigenin,” “antihypertensive,” and “hypotensive.” The search was limited to articles published between January 2013 and December 2023. The inclusion criteria were original research articles that involved both animal and human subjects, published in English, and reported results applicable to the subject of this review. Review articles or articles in the form of theses or books were excluded.

### 2.1. Data Extraction

**Figure 1:**
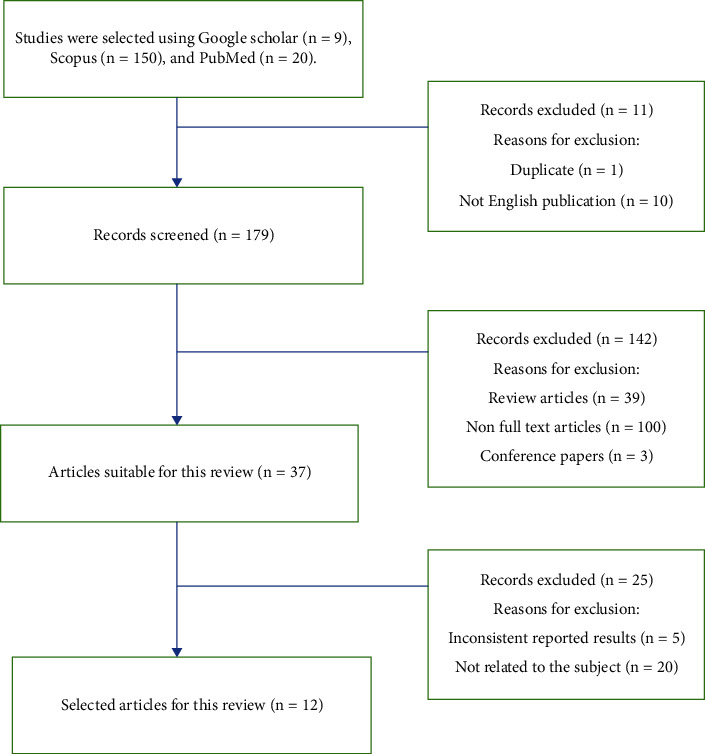
Selection procedure of the studies. n: number.

Titles and abstracts of 179 studies included in the search were analyzed by two independent reviewers. Subsequently, full-text articles were read to determine whether they qualified for inclusion in the review, and only 12 studies related to the hypotensive effect of celery were extracted. Information about the study design, sample size, celery administration protocols, and outcomes was extracted from eligible studies (Figure 1).

### 2.2. Data Synthesis

Narrative synthesis was performed to collect and summarize the extracted studies. The results of these studies were classified as animal or human studies. A description of each study's design, sample size, intervention strategy, and findings is included in the synthesis. For this narrative review, both authors individually gathered information from each manuscript to minimize potential biases and errors. Subsequently, they collectively agreed on the final selection of the studies to be included.

## 3. Results

### 3.1. Literature Search

Twelve articles regarding the effectiveness of celery on hypertension were obtained from the database and included in this narrative review. Five articles were on animals and seven were on humans.

### 3.2. Effect of Celery and Its Extracts on Hypertension

Five experimental studies that used animal models to study the effects of celery on hypertension were included in this review. Of those trials, four studies demonstrated that celery and its different extracts caused a significant decrease in blood pressure in a hypertensive study model [15–18]. However, Tashakori-Sabzevar et al. [15] reported that the hypotension effect of celery was found not only in hypertensive groups but also in normotensive groups [15]. Another study showed that celery extract has a vasorelaxant effect on rat aortic rings, which has a positive impact on BP, but there was no measurement of BP reduction [19].

It is noteworthy that four of the included animal studies were controlled trials. Studies have used different control models such as tap or distilled water [17, 18], hypertension-lowering agents (spironolactone [16] and nifedipine [15]), and induced-hypertension models such as fructose-induced hypertension [17], liquid paraffin and normal saline [16], and NaCl with prednisone [18].

Moghadam et al. [16] and Tashakori-Sabzevar et al. [15] analyzed the n-butylphthalide content using different extraction methods. They found that hexanic celery extract had a higher n-butylphthalide content than methanolic [16] or ethanolic extracts [15, 16].

Regarding the effect of celery on heart rate, studies in this review reported different heart rate responses after celery treatment, ranging from increased heart rate [16], decreased heart rate [15], and no alteration [17].

Rosa and Rivai [18] showed the effect of combining celery with another medicinal plant, garlic. The results demonstrated a significant reduction in the BP. However, this result was heavily influenced by the dose and duration of administration of this combination [18].

Seven interventional trials conducted in humans were included in this review. All of these trials confirmed that celery had a positive effect on hypertension. Statistically, six studies showed a significant reduction in BP after celery administration [9, 12, 20–23]. One trial was a case report of an elderly man with chronic hypertension who experienced uncommon side effects from hypertension medications. Administration of celery juice in his treatment regimen for six months resulted in a positive response and a reduction in SBP by 32 mmHg [13].

All included studies in this review, with the exception of case report one, were controlled trials using either placebo [12, 20–22] or no treatment groups [9, 23] to evaluate the effect of celery.

Administration of 250 mg of celery stem extract reduced 9.59 mmHg in SBP and 15.2 mmHg in DBP [23]. Also, intervention with 1.34 g of celery seed extract showed a reduction of 11 mmHg in SBP and 8 mmHg in DBP [22]. These results confirm the positive effect of celery extract on hypertension.

A BP reduction of 11 mmHg in SBP was shown after administration of ethanolic celery seed extract [12, 20, 22]. A reduction in SBP of 17 mmHg was found after celery juice treatment [9], and a reduction of 19 mmHg was found after ethanolic celery stem extract [23]. This indicates that the extraction method and plant parts influence the effect of celery on the BP.

In terms of the safety of using celery as a supplement, Gautam [20] reported that celery seed extract is safe and well tolerated by patients with hypertension [20]. In addition, Shayani Rad et al.'s [22] study showed that the results of safety parameters demonstrated that celery seed extraction is safe and beneficial in different biochemical parameters [22].  Table 1 summarizes studies on the hypotensive effects of celery.

### 3.3. Underlying Mechanism of Action of Antihypertension Bioactive Compounds of Celery in Hypertension

Hypertension is a serious health problem and a factor that worsens other cardiovascular diseases. Also, it is a principal cause of premature mortality. Regarding the pathophysiology of hypertension, there are many effectors involved. Potassium channels, renin-angiotensin system, nitric oxide, calcium ions, and reactive oxygen species are the most important effectors that regulate the tone of the vascular system. Therefore, any disruption of one of these effectors may lead to hypertension [24].

Although there are several types of hypertension-lowering pharmaceutical agents, natural sources have inspired many compounds that can modulate the hypertension pathway with minimal side effects. One of the natural plants traditionally used to lower BP is celery or other types of celery extracts (stem, seed, and leaves) [6]. Various plant parts have been used in extraction and medical applications. Celery contains many bioactive compounds that have a positive effect on hypertension reduction [6].

Data from in vitro and in vivo animal studies have demonstrated that celery exerts beneficial effects on hypertension. In rats with hypertension, ethanolic celery seed extracts (0.05, 0.1, 0.25, 0.5, 1, and 2 mg/ml) exerted a vasorelaxant effect on rat aortic rings. The possible mechanisms of action include calcium influx blocking into smooth muscle cells and voltage-dependent potassium channel activation [19]. One of the most bioactive compounds of celery is n-butylphthalide, and it can also exert a beneficial effect on lowering the blood pressure. Hexanic extract of celery contains 3.7-4 more times of n-butylphthalide, and at a dose of 300 mg/kg, the extract can decrease SBP by about 38 mmHg [16]. Also, other bioactive compounds in celery play a role in BP reduction, such as high expression of angiotensin-converting enzyme 2 by apigenin [25], vasodilation by linalool [26], and inhibition of angiotensin II proliferation and migration by luteolin [27].

The complete mechanism of action by which celery and its compounds can regulate blood pressure is not clearly understood in the literature. However, it is now possible to postulate its proposed mechanism(s) of action on blood pressure.  Table 2 summarizes the mechanism of action of the possible hypotensive bioactive compounds in celery and different parts of the plants.

## 4. Discussion

The current narrative review provides the available evidence from animal and human intervention trials evaluating the effect of celery on hypertension in the last decade. Collectively, the results of this literature search showed a considerable number of studies on the topic. The potential effect of celery on hypertension has been extensively discussed. Celery is a medicinal herb, and it has been widely reported to possess an antihypertension property. However, recent scientific data suggest semisimilar results in human and animal studies, with little quantitative difference in responses and BP reduction. The possible reason for the different results may be due to the use of different doses, duration of intervention, and celery species, in addition to different administration forms (whole plant or extract's capsule). Moreover, some other studies have assessed the influence of celery, and they reported that it could also be affected by the method of extracting and the extracted plant part. It is worthy to note that the effect of celery and its extracts is considered to be dose dependent in addition to the influence of the duration of administration.

In hypertensive patients, the SBP and DBP could be regulated with celery administration. This property of celery is assumed to play an essential role because hypertension is the main factor that worsens other cardiovascular diseases, and it is considered the silent killer [35].

Celery extracts contain different bioactive compounds that aid in BP regulation. Each of these compounds exerts its effect through different pathways. Recent studies have focused on the n-butylphthalide content during extraction [15, 16]. This compound exerts its effect via diuretics and vasodilation and decreases oxidative stress and the expression of IL-6, TNF-*α*, and NF-*κ*B [16, 28, 29].

Other possible bioactive compounds responsible for the hypotensive effect could be *Apium graveolens* [31], n-butylphthalide [16, 28, 29], D-limonene [32], apigenin [30], linalool [26], and luteolin [27]. Nevertheless, using the whole plant or part of it also showed a positive effect on BP [1, 15, 17, 34].

Various studies have explored the hypotensive effect of celery, with a focus on its molecular mechanism. Tashakori-Sabzevar et al. [15] demonstrated that one of the possible mechanisms of the hypotension effect is a blocking of calcium influx into cells or calcium release from the sarcoplasmic network that is found in smooth muscles [15]. In addition, the nitric oxide pathway is involved in celery's vasorelaxant effect. However, disruption of the endothelium could effectively decrease the vasorelaxant effect of celery, indicating that nonendothelial pathways are more likely to be involved in this effect [15]. In contrast, Sohrabi et al. [19] reported an endothelium-dependent pathway in the preventive vasodilation effect of celery. The indication for this finding was the pretreatment of aortic rings with indomethacin or L-NAME, which did not prevent the vasodilation effect of celery, indicating the involvement of other endothelium-dependent pathways in celery vasodilation [19]. In addition, Branković et al. [36] further demonstrated that both aqueous and ethanol extracts of celery can lower blood pressure and reduce the contractility of isolated atria, potentially mediated by muscarinic receptor stimulation [36]. A recent molecular study conducted by Ma et al. [37] revealed that many components in celery seeds play main roles in the ACE inhibitory activity including flavonoids, terpenoids, and carbohydrates. It is worth noting that ultrafiltration technique in this study increases ACE inhibitory activity of celery seed aqueous extract [37].

Further studies should be conducted in order to understand the long-term effects of celery supplementation. Also, identification of the most bioactive compounds in celery exerts the antihypertensive effect and their complete mechanism of action. The dose-response relationship and duration must be studied. More studies are needed in the context of the safety of the chronic use of celery as a daily routine for hypertensive patients.

## 5. Conclusion

Hypertension is increasing worldwide and contributes to different cardiovascular diseases. Targeted celery-based interventions can provide an opportunity to regulate high blood pressure to avoid progression to other complications. Similar results have been reported in scientific literature. Thus, celery can be considered an antihypertensive agent. However, a dose-response relationship should also be studied, as it is a major factor in disease prevention and treatment strategies.

## Figures and Tables

**Table 1:** Summary of studies on the hypotensive effects of celery.

Author	Total (*n*)	Gender (*n*)		Age	Study design	Celery administration				Results		
		Male	Female			Administration form		Dosage	Duration	Decrease in blood pressure (mmHg)		Hypertension-related key findings
						Control group	Intervention group			Systolic (mmHg)	Diastolic (mmHg)	
Animal studies												
Moghadam et al. [16]	54	54	—	N/A	Experimental	G6 (PC): spironolactone G7, 8 (NC): NS 0.9% and liquid paraffin	G1, 2, 3: hexanic extract G4: methanolic extract G5: aqueous-ethanolic extracts G9: NS 0.9% and hexanic extract	G1: 100 mg/kg G2: 200 mg/kg G3, 4, 5: 300 mg/kg G9: 0.5 ml of NS (0.9%), then 300 mg/kg	3 weeks	G3: 38 mmHg G4: 24 mmHg G5: 23 mmHg	N/A	(i) All types of celery extracts cause a reduction in SBP and increased HR in hypertensive groups but not the normotensive group (ii) Hexanic extract contains 3.7 to 4 more n-butylphthalide content than methanolic and aqueous-ethanolic extract, respectively
Sohrabi et al. [19]	105	105	—	N/A	Experimental	—	Ethanolic celery seed extract	0.05, 0.1, 0.25, 0.5, 1, and 2 mg/ml	N/A	—	—	(i) Celery seed extract has endothelium-dependent vasorelaxant effects in rat's aortic rings (ii) There are two possible relaxation mechanisms: inhibition of calcium influx into smooth muscle cells and activation of voltage-dependent potassium channels
Tashakori-Sabzevar et al. [15]	30	30	—	N/A	Experimental	Nifedipine (calcium blocker agent)	Hexanic extract	2.5, 5, 7.5, 10, 12.5 mg/kg	N/A	Dose 12.5 mg/kg: 69.5% reduction in normotensive groups and 34.6% in hypertensive groups	Dose 12.5 mg/kg: 80% reduction in normotensive groups and 37.6% in hypertensive groups	(i) Hexanic extract contains more n-butylphthalide than aqueous-ethanolic extract (ii) Hexanic celery extract significantly reduces SBP, DBP, MABP, and HR in normotensive and hypertensive groups (iii) Involved mechanisms in celery's hypotensive effect are vasodilation effect via blocking calcium channels and bradycardia induction (iv) Celery's hexanic extract and nifedipine have a similar effect on BP but not HR
Dianat et al. [17]	40	40	—	N/A	Experimental	1: tap water 2: fructose extract	Methanol celery leaf extract	100 and 200 mg/kg	7 weeks	N/A	N/A	(i) There is a significant reduction in SBP in groups receiving fructose and celery extract in comparison with fructose group (ii) No significant HR alteration in any group
Rosa and Rivai [18]	25	25	—	2-3 months	Experimental	NC: distilled water PC: 8% NaCl and 0.05% prednisone	Celery (whole plant)+garlic (whole)	185 mg/200 g BW, 370 mg/200 g BW, 740 mg/200 g BW	3 weeks	Dose 185 mg 35.16 Dose 370 mg 26.71 Dose 740 mg 45.44	Dose 185 mg 34.73 Dose 370 mg 28.16 Dose 740 mg 38.85	(i) Hypotension effect of combined celery and garlic is significant, and it relies heavily on dose and duration of the treatment
Human studies												
Azizah et al. [9]	24	16	8	40-50 years old	Quasiexperiment using the form of pretest and posttest control group design	No treatment	Celery juice	N/A	N/A	17.58	7.08	(i) Significant effect of celery juice on hypertension patients was found (ii) Celery juice has a strong tolerance to SBP and moderate closeness to DBP
Gautam [20]	N/A	N/A	N/A	N/A	Crossover placebo controlled	Placebo	Celery seed extract	N/A	N/A	11.17	8.005	(i) Significant decrease in BP was found with difference related to gender. Females show a higher response in lowering BP
Ahmad and Rahman [21]	36	9	27	>55 years old	Quantitative experimental study using the form of pretest and posttest nonequivalent control group design	Placebo	Celery extract	N/A	30 days	12.7	7.7	(i) Celery extract has a significant reduction in SBP and DBP in elderly with hypertension
Shayani Rad et al. [22]	52	26	26	29-63 years old	Randomized, triple-blind, placebo-controlled, crossover, clinical trial	Placebo	Ethanolic celery seed extract	1.34 g extract/day	4 weeks	11	8	(i) There is a significant decrease in both SBP and DBP after celery extract treatment (ii) Celery is considered safe for hypertension patients with positive effect on BG and lipid profile (iii) There is no change in HR in both control and intervention groups
Zafar et al. [23]	30	—	30	18-45 years old	Controlled randomized trial	No treatment	Ethanolic celery stem extract	250 mg/day	60 days	19.59	15.12	(i) There is a significant decrease in both SBP and DBP after celery extract treatment (ii) There is a notable change in HR
Shayani Rad et al. [12]	51	26	25	20-70 years old	Triple-blind, placebo-controlled, crossover	Placebo	Ethanolic celery seed extract	1.34 g extract/day	4 weeks	11.08	6.54	(i) There is a significant decrease in both SBP and DBP after celery extract treatment
Illes [13]	1	1	—	74 years old	Case report	—	Celery juice	10-12 stalks	6 months	32	N/A	(i) Positive response to include celery juice into daily regimen

Abbreviations: N/A: not applicable; G: group; PC: positive control; NC: negative control; NS: normal saline; BP: blood pressure; SBP: systolic blood pressure; DBP: diastolic blood pressure; HR: heart rate; BG: blood glucose; MABP: mean arterial blood pressure.

**Table 2:** Summary of antihypertensive bioactive compounds in celery and their mechanism of action.

Bioactive compound	Effect	Mechanism	Evidence
3-n-Butylphthalide	Antihypertension effect	(i) Voltage- and receptor-operated calcium channel blocking (ii) Decreasing oxidative stress and expression of IL-6, TNF-*α*, and NF-*κ*B (iii) Vasodilation (iv) Diuretics	[16, 28, 29]
Apigenin	Antihypertension effect	(i) Overexpression of angiotensin-converting enzyme 2 (ii) Blocking of calcium channel blocker	[25, 30]
*Apium graveolens*	Antihypertension effect	(i) Antagonist to calcium channel	[31]
D-limonene	Antihypertension effect	(i) Antioxidant	[32]
Linalool	Antihypertension effect	(i) Vasodilator	[26]
Luteolin	Antihypertension effect	(i) Inhibition of the proliferation and migration of angiotensin II	[27]

*Plant parts*			
Celery juice	Antihypertension effect	(i) Receptor-operated calcium channel blocking	[33]
Leaf	Antihypertension effect	(i) Vasodilator	[17]
Seed	Antihypertension effect	(i) Calcium channel blocker (ii) Vasodilator (iii) Decreased heart rate	[15]
Root	Antihypertension effect	(i) Decrease level of angiotensin II	[34]

Abbreviations: IL-6: interleukin-6; TNF-*α*: tumor necrosis factor-*α*; NF-*κ*B: nuclear factor kappa B.

## Data Availability

The datasets used and/or analyzed during the current study are available from the corresponding author upon reasonable request.
